# *Dissertations into Practice*: 10 years on, 40 articles later

**DOI:** 10.1111/hir.12422

**Published:** 2022-02-07

**Authors:** Frances Johnson

**Affiliations:** ^1^ Department of Languages, Information and Communication Manchester Metropolitan University Manchester UK

**Keywords:** dissemination, dissertations, education, graduate, librarians, students

## Abstract

Frances Johnson, Regular Feature Editor, looks back on the first 10 years of student projects published in *Dissertations into Practice*. Forty articles later, she notes that the value of these short features, then as now, is in recognising the potential of student research to a wider audience and the implications of their research on practice.

## INTRODUCTION

Ten years ago, this feature *Dissertations into Practice* was launched. The purpose was two‐fold: to encourage students and new professionals to write for publication and highlight the potential impact of student research on policy and practice. The introduction to the feature, outlining the rationale, was published in the March 2012 issue (*Health Information and Libraries Journal*, **29**(1), 72–74).^1^ Then, as now, the scope was intentionally broad, in that any dissertation topic and approach can be considered for inclusion, provided it is related to the provision of health information or health library services.

Ten years on, 40 feature articles have been published, the majority of which have come from recently graduated students from Library and Information Science courses (or related, such as Health Informatics) based on a recently completed dissertation for Masters. On occasion, an article has been adapted from a PhD thesis. Regardless of the broad scope, the reason why the feature has been successful, year in and year out, can be explained by the three main contributors to this achievement so far: the student (or graduate), their dissertation supervisor and their audience, the readership of the *Health Information and Libraries Journal*, and this feature. In most instances, students and new professionals are supported by their supervisor and the feature editor to write for their first publication and highlight the potential impact of their research for practice. Academic staff in these university departments, who each year supervise students to complete a dissertation, are keen to see work from good dissertations highlighted in this way through publication. And, this is key point of *Dissertations into Practice*; these student research projects have value to a wider audience, especially when all contributors to the feature are encouraged to emphasise the implications of their research on practice.

## CORE THEMES OF PUBLISHED ARTICLES

Given the broad scope, the range of contributions to the feature so far has been diverse. For all submissions, authors are required to concentrate on four main aspects: the context, that is, why the topic was chosen, the research methods used, the key findings, and the implications for practice. With the emphasis on the implications for practice most are, indeed, practical in nature, rather than theoretical, and relate to a range of sectors for the delivery of health information, for example, public, academic, and health libraries. Papers published since inception tend to report on studies that evaluate existing services such as health information provision by public libraries or, for example, investigate service improvements, such as access to electronic journals in NHS libraries or outreach services. These studies may seek to explore the opinions of stakeholders or investigate the challenges, as well as the need to measure the impact of such services. The measurement of impact of libraries and information services is essential yet rarely straightforward, and these small‐scale studies can often point to interesting and insightful approaches. Dissertations relating to health information will often investigate the information (seeking) behaviour of the people, that is, the public, patients, medics, and allied health professionals and workers. Why do people seek health information, and what resources do they use? The use of technology to enhance services is also a common theme—unsurprising given the range of digital innovation used for the provision of health information. Furthermore, these studies can often lead to recommendations for implementing change and/or an evaluation leading to improved design for access, as well as focusing on health information services and student dissertations, and the papers published here have explored the role of the information professional, not least in providing information literacy skills training, and its advocacy within the organisation, as well as in conducting searches for systematic reviews and evidence bases.

## TEN YEARS OF *DISSERTATIONS INTO PRACTICE*

The article titles from March 2012 to this issue are listed here to give a sense of the interesting, valuable, and timely dissertations and research projects that are influencing policy and practice. Together, they build into a valuable resource in itself and for the development and enhancement of excellence in all aspects of health information provision and its services. If you are interested in being supported to share your work as part of this diverse collection of student health information or health library service projects, please do get in touch; contact details above.

2012 (Volume 29)
  - Implementing RFID in a hospital library: a scoping study
  - An evaluation of a books on prescription scheme in a UK public library authority
  - Health information seeking in the information society.
2013 (Volume 30)
  - The NHS Lanarkshire Intranet site (FirstPort) and its effectiveness as a knowledge management tool.
  - An evaluation on the effectiveness of Web 2.0 Startpages (Netvibes&Page flakes) within NHS libraries.
  - An investigation into the move towards electronic journals: a case study of NHS libraries Kent, […]
  - Optimising the retrieval of information on adverse drug effects.
2014 (Volume 31)
  - Outreach services in healthcare libraries: perceptions and impacts.
  - Can your public library improve your health and well‐being?
  - Health care librarians and information literacy: an investigation
  - An investigation into the feasibility of designing a framework for the quantitative evaluation of the clinical librarian service at an NHS Trust in Brighton
2015 (Volume 32)
  - HIV/AIDS‐related stigma and information behaviour: an ethnographic study in the UK.
  - The health information seeking behaviour and needs of community health workers in Chandigarh in Northern India.
  - Addressing Library Anxiety (LA) in student nurses: a study in an NHS Foundation Trust Hospital library
2016 (Volume 33)
  - Can Twitter improve your health? An analysis of alcohol consumption guidelines on Twitter
  - Barriers to the use of the library service amongst clinical staff in an acute hospital setting
  - Empowering international nursing students to become effective library users
  - Exploring trust in online health information: a study of user experiences of patients.co.uk
2017(Volume 34)
  - The role of information therapy reducing anxiety in patients undergoing *in vitro* fertilisation treatment
  - The information needs of occupational therapy students: a case study
  - The impact of body image on Fitbit use: a comparison across genders
2018 (Volume 35)
  - eBook management in NHS libraries in the North of England: perceptions and practice
  - Proposed information outreach programme in primary and secondary health care of Punjab, Pakistan
  - Professional collaboration in searching the evidence for an ill‐defined concept
  - Rethinking bibliotherapy: a neurorhetoric narratology model for addiction treatment
2019 (Volume 36)
  - Electronic healthcare records and data quality
  - The impact of orphan drug policies in treating rare diseases
  - Health information seeking behaviour: the librarian's role in supporting digital and health literacy
  - Discussing teamwork in health care: from interprofessional collaboration to coordination and cooperation
2020 (Volume 37)
  - Information seeking patterns of psychiatrists during clinical practice
  - Use of bibliometric analysis of Greek hospital personnel publications
  - Library Jargon Creates Barriers for Potential Users of Health Library and Information Services
  - Mapping a high‐level overview of information flows in the Dutch declaration chain for medical specialist health care
2021 (Volume 38)
  - Outreach marketing may be a successful strategy for NHS libraries
  - Delivering eye health education to deprived communities in India through a social media‐based innovation
  - Algorithmic literacy in medical students—results of a knowledge test conducted in Germany
  - Library research support services in China’s universities of traditional medicine: Understanding user requirements

## ENDNOTE

^1^
Marshall, A. Calling all students!!!. Health Information and Libraries Journal 2012, 29, 72–74.

